# Evening Primrose Extracts Inhibit PDGF-BB-Induced Vascular Smooth Muscle Cell Proliferation and Migration by Regulating Cell-Cycle-Related Proteins

**DOI:** 10.3390/cimb44050131

**Published:** 2022-04-27

**Authors:** Jin-Ho Lee, Min Jeong Kim, Keun-Jung Woo, Joonpyo Hong, Sun-Hong Kim, Tack-Joong Kim

**Affiliations:** 1 Division of Biological Science and Technology, Yonsei University, Wonju 26493, Korea; drlogos@naver.com (J.-H.L.); aw8596@naver.com (M.J.K.); rmswjd@yonsei.ac.kr (K.-J.W.); hhjoonpyo@naver.com (J.H.); 2 Research & Development Center, Doctor TJ Co., Ltd., Wonju 26493, Korea; auscrlt1028@gmail.com

**Keywords:** evening primrose, *Oenothera biennis*, vascular smooth muscle cell, cardiovascular disease

## Abstract

The proliferation and migration of vascular smooth muscle cells (VSMCs) are important factors in the occurrence of cardiovascular diseases, such as blood flow abnormalities, stroke and atherosclerosis. Evening primrose, known as *Oenothera biennis*, is a plant native to Korea that exerts physiological activities, such as antioxidant effects, the inhibition of lipid accumulation and the prevention of muscle atrophy. However, the function of evening primrose stem (EVP) in the regulation of VSMC proliferation and migration and the underlying mechanisms have not been identified. In this study, the effect of EVP on the platelet-derived growth factor (PDGF)-induced proliferation and migration of VSMCs was investigated. The results show that PDGF-BB-induced proliferation of VSMCs was inhibited by EVP at concentrations of 25, 50 or 100 μg/mL in a concentration-dependent manner, and a migration assay showed that EVP inhibited cell migration. Cell cycle analysis was performed to confirm the mechanism by which cell proliferation and migration was inhibited. The results indicate that proteins involved in the cell cycle, such as cyclin, CDK and phosphorylated Rb, were downregulated by EVP at concentrations of 100 μg/mL, thereby increasing the proportion of cells in the G0/G1 phase and inhibiting cell cycle progression. In the PDGF receptor (PDGFR) signaling pathway, phosphorylation of the PDGFR was inhibited by EVP at concentrations of 100 μg/mL, and PLCγ phosphorylation was also decreased. The PDGF-BB-induced effect of EVP on the proliferation of VSMCs involved the inhibition of Akt phosphorylation and the reduction in the phosphorylation of MAPK proteins such as ERK, P38 and JNK. In conclusion, the results demonstrate that EVP inhibited PDGF-BB-induced VSMC proliferation and migration by regulating cell-cycle-related proteins.

## 1. Introduction

It is not only older adults who are at risk of vascular disease but also younger individuals who do not participate in physical activities and consume a high-calorie diet. Vascular diseases occur when smooth muscle cells proliferate abnormally, leading to blockage of blood vessels in the endothelium [1]. Disturbed blood flow is a major cause of chronic diseases such as blood flow abnormalities and stroke. These conditions are associated with myocardial infarction and atherosclerosis of the heart, which lead to higher mortality rates and decreased life activities [2,3]. Vascular smooth muscle cells (VSMCs), one of the major cell types that form the vascular wall, can develop arteriosclerotic lesions during proliferation [4,5]. In particular, as expression levels of platelet-derived growth factor (PDGF) increase in atherosclerotic lesions, PDGF-induced increases in mitosis and proliferation cause vasodilation after angiogenesis [6].

PDGF is one of the main regulatory factors that drives mitosis in VSMCs [7]. VSMC proliferation is induced by polypeptides and growth factors in the blood, such as PDGF, transforming growth factor β (TGF-β) and angiotensin (Ang) [8,9,10]. PDGF contributes to the proliferation and migration of VSMCs through intracellular PDGF-induced phosphorylation cascade and mediates many biological effects [11]. Thus, studies on the inhibition of PDGF-induced VSMC proliferation and migration provide insight into the phased prevention of atherosclerosis [12].

The proliferation of VSMCs is initiated by proliferation signals and is regulated by the cell cycle [13]. The cell cycle proceeds in three phases (G0/G1, S and G2/M), and molecular markers such as cyclin, CDK, Rb and p27 regulate the progression through each phase [14]. Complexes are formed by cyclin D and CDK4, as well as cyclin E and CDK2, and both complexes are required to transition from the G1 phase to S phase of the cell cycle [15]. The downregulation of phosphorylated Rb induces G1 arrest [16].

Evening primrose has antioxidant properties and regulates intracellular signaling proteins. In particular, the seeds have a high phenolic content and have attracted attention due to their antioxidant properties [17]. In addition, the evening primrose root exerts protective and recuperative effects against damage from reactive oxygen species (ROS) in muscle cells [18]. Importantly, evening primrose stem (EVP) extracts have been reported to be effective in anti-inflammatory, anti-diabetic and anthelmintic activity [19]. However, the effects of EVP on the proliferation and migration of PDGF-BB-induced VSMCs have not been reported to date. Therefore, the effect of EVP on PDGF-BB-induced VSMC proliferation and migration was investigated in the present study using signaling pathway and cell cycle analysis.

## 2. Materials and Methods

### 2.1. Preparation of Evening Primrose Stem Extract

Evening primrose was obtained from the Rural Development Administration of the Republic of Korea. The evening primrose stem (EVP) was dried and ground. After adding 1.0 L of distilled water to 100 g of the pulverized EVP sample, the mixture was placed in a hot bath at 100 ± 5 °C for 2–3 h. The boiled sample was cooled to a constant temperature to prevent loss from evaporation. The obtained EVP extract was filtered under reduced pressure and freeze-dried for future experiments.

### 2.2. Material

PDGF-BB was purchased from KOMA BIOTECH Inc. (Seoul, Korea). The primary antibodies used in this study were against CDK2, CDK4, cyclin E, Akt, P-Akt (Ser473), PLC-γ, P-PLC-γ (Tyr783), ERK1/2, P-Erk1/2 (Thr202/Tyr204), p38, P-p38 (Tyr182), JNK, P-JNK (Thr183/Tyr185), P-Rb (Ser807/Ser811), P-PDGFR (Tyr1021), β-actin (Cell Signaling Technology, Danvers, MA, USA) and cyclin D1 (Santa Cruz Biotechnology, Dallas, TX, USA). The secondary antibodies used were anti-rabbit and anti-mouse (Cell Signaling Technology, Danvers, MA, USA).

### 2.3. Cell Culture

Vascular smooth muscle cells (VSMCs) were purchased from American Type Culture Collection (ATCC, Manassas, VA, USA) and maintained in Dulbecco′s Modified Eagle′s Medium (DMEM) supplemented with 10% FBS, 1% penicillin and 1% streptomycin (Sigma-Aldrich, St. Louis, MO, USA). The cells were cultured at 37 °C in an incubator with 5% CO_2_. VSMCs were seeded in a complete medium for 24 h.

### 2.4. Cell Proliferation Assay

The proliferation of VSMCs was evaluated using an MTT assay (Duchefa Biochemie, Haarlem, The Netherlands), according to a previously published protocol [20,21]. VSMCs (1.0 × 10^4^ cells/well) were seeded in 24-well plates and cultured in fresh serum-free medium for 22 h. The cells were incubated with or without EVP (25, 50, or 100 μg/mL). After 2 h, the medium was replaced with fresh serum-free medium containing 50 ng/mL PDGF-BB, and cells were incubated for 24 or 48 h. After the experimental treatment, 150 μL of MTT (thiazolyl blue) solution was added to each well. The plates were then incubated for 2 h at 37 °C. Absorbance was measured at 595 nm using an ELx800 microplate reader (BioTek, Basel, Switzerland).

### 2.5. Cell Migration Assay

The protocol used to assess cell migration was based on Frontier’s method [22]. Cell migration assays were performed in 6-well plates. When cells reached 90% confluence, the medium was replaced with fresh serum-free medium for 22 h. A single wound was then created in the center of the cell monolayers by gentle removal of the attached cells with a sterile plastic pipette tip. After 24 or 48 h of incubation, cells that had migrated into the wounded area or protruded from the border of the wound were visualized and photographed with a DP80 inverted microscope (Olympus, Tokyo, Japan).

### 2.6. Cell Cycle Analysis Based on Flow Cytometric Analysis

VSMCs were harvested, fixed in 100% ethanol at 4 °C, then washed twice with PBS and incubated with 10 μg/mL RNase A (Invitrogen, Waltham, MA, USA) and 50 μg/mL propidium iodide solution for 30 min at 37 °C. Fluorescence from 1.0 × 10^4^ cells was captured using flow cytometry (BD Biosciences, Franklin Lakes, NJ, USA). The percentage of cells in the G0/G1, S and G2/M phases of the cell cycle was calculated using flow analysis software (Turku Bioscience, Turku, Finland).

### 2.7. Western Blot Analysis

The cells were lysed in lysis buffer (iNtron Biotechnology, Seongnam, Korea). After incubation on ice for 30 min, the samples were harvested. Protein samples were separated on 8–15% SDS-polyacrylamide gels and transferred to PVDF membranes (Bio-Rad Laboratories, Hercules, CA, USA) using a standard electrophoretic procedure. The membranes were incubated with primary antibodies overnight at 4 °C, and goat-conjugated secondary antibodies were used for detection. Antibody treatment was performed with primary antibodies (CDK2, CDK4, cyclin D1, cyclin E, P-Rb, Rb, P-PLC-γ, PLC-γ P-ERK1/2, ERK1/2, P-JNK, JNK, P-Akt, Akt, P-PDGFR, PDGFR and β-actin) at a ratio of 1:2500. Membranes were washed three times with Tris-buffered saline solution containing Tween 20 for 10 min, and then secondary antibodies were added at a ratio of 1:5000 for 2 h at room temperature. Signals were detected using an LAS detection system (GE Healthcare, Marlborough, MA, USA), and the signal intensity was quantified using ImageJ software (National Institutes of Health, Bethesda, MD, USA).

### 2.8. Statistical Analysis

The experimental results are expressed as mean ± standard error (SE). ANOVA and paired or unpaired *t*-tests were performed as appropriate. Statistical significance was set at *p* < 0.05. All experiments were performed at least three times.

## 3. Results

### 3.1. EVP Inhibits PDGF-BB-Induced VSMC Proliferation and Migration

**Figure 1:**
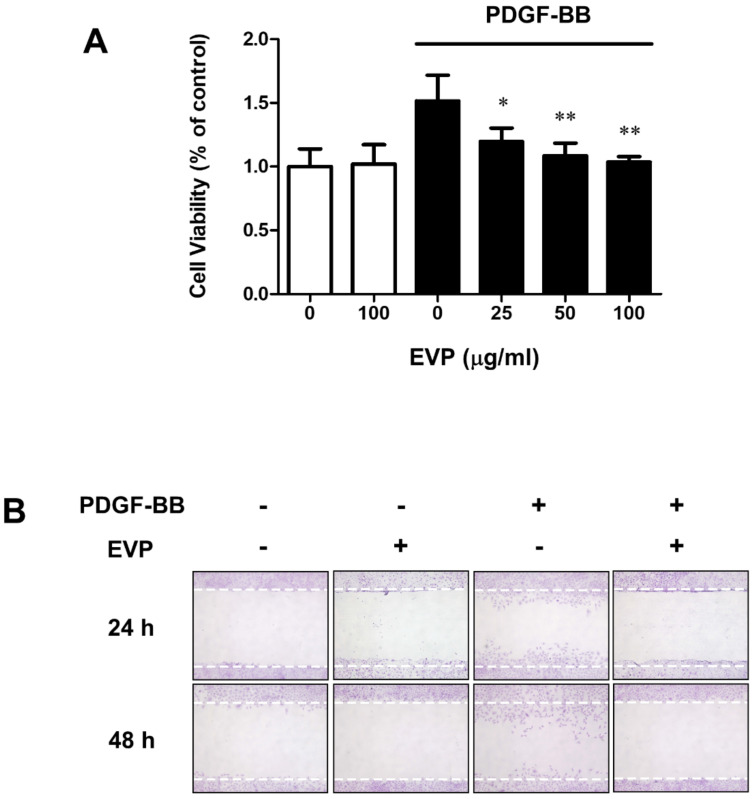
Effect of EVP on PDGF-BB induced VSMC proliferation and migration. (**A**) EVP inhibited PDGF-BB-induced proliferation of VSMCs. The cells were pre-cultured in serum-free medium for 22 h and then treated with various concentrations of EVP in serum-free medium for 2 h. The cell viability of each group with or without EVP treatment was measured using an MTT assay 48 h after stimulation with 50 ng/mL PDGF-BB. (**B**) EVP inhibited PDGF-BB-induced migration of VSMCs. The cells were pre-cultured in serum-free medium for 22 h, then treated with various concentrations of EVP in serum-free medium for 2 h. Confluent VSMCs were wounded by scraping and allowed to migrate for 24 and 48 h. Data are expressed as mean ± S.E.M. (*n* = 4); * *p* < 0.05, ** *p* < 0.01 compared with PDGF-BB-only treated group.

We measured the viability of VSMCs according to EVP concentration to determine the effect of EVP on the proliferation of PDGF-BB-induced VSMCs. Additionally, a migration assay was performed to confirm the effect of EVP on cell migration and proliferation. The effect of EVP on the PDGF-BB-induced proliferation of VSMCs was examined using an MTT assay. VSMCs were pretreated with EVP (25, 50 or 100 μg/mL) in serum-free medium for 48 h and then stimulated with PDGF-BB (50 ng/mL) for 48 h. PDGF-BB treatment significantly increased the proliferation of VSMCs compared with that of untreated cells (1.52 ± 0.20). PDGF-BB-induced VSMC proliferation was significantly decreased by EVP at concentrations of 25 (1.20 ± 0.11), 50 (1.09 ± 0.10) and 100 μg/mL (1.03 ± 0.04). In addition, EVP treatment in the absence of PDGF-BB did not decrease the viability of VSMCs compared with that of untreated cells (1.02 ± 0.15) (Figure 1A).

To measure the effect of EVP on VSMC migration, a cell migration assay was performed and migration after treatment with EVP was observed. As shown in Figure 1B, treatment with PDGF-BB (50 ng/mL) promoted VSMC migration. Stimulation with PDGF-BB for 24 and 48 h increased the number of migrated VSMCs, but treatment with 100 μg/mL EVP significantly reduced the number of migrated cells (Figure 1B). These results indicate that EVP inhibited PDGF-BB-induced VSMC proliferation and migration.

### 3.2. Dose-Dependent Effects of EVP on the Cell Cycle in PDGF-BB-Induced VSMCs

The effects of EVP treatment on cell cycle progression in PDGF-BB-induced VSMCs were investigated using flow cytometry. VSMCs (75.79%) were synchronized in the G0/G1 phase of the cell cycle for 24 h. The proportion of VSMCs in the G2/M phase increased (from 11.45% ± 0.50 to 34.61% ± 0.53) and that in the G0/G1 phase decreased after induction with PDGF-BB, indicating that PDGF-BB triggered cell proliferation.

**Figure 2:**
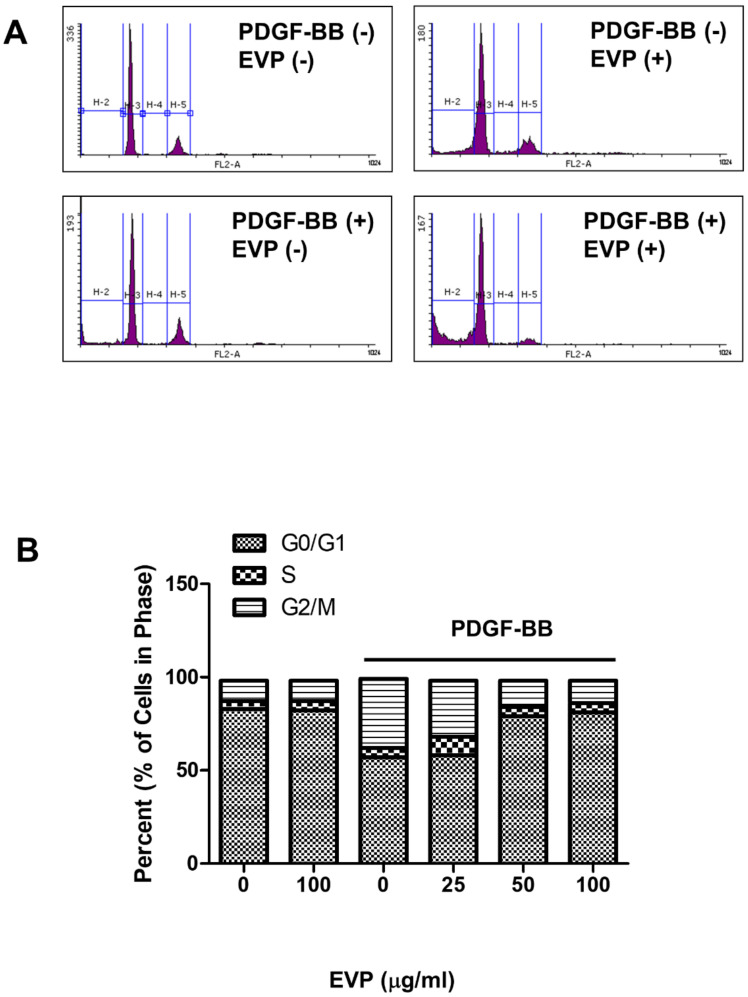
Effect of EVP on the cell cycle in PDGF-BB-induced VSMCs. Cell cycle progression was assessed by flow cytometric analysis of DNA. The cells were pre-cultured in serum-free medium for 22 h and then treated with various concentrations of EVP in serum-free medium for 2 h. (**A**) Histograms depicting the effect of EVP on the cell cycle. Cells were treated with 100 μg/mL of EVP, followed by stimulation with 50 ng/mL PDGF-BB. After 24 h, the quantity of nuclear DNA in each sample was reflected by the fluorescence intensity of incorporated propidium iodide. (**B**) The effect of EVP on the proportion of cells in each phase of the cell cycle. Cells were treated with EVP at various concentrations (0, 25, 50 and 100 μg/mL) and then treated with 50 ng/mL PDGF-BB for 24 h. The quantity of nuclear DNA in each sample was reflected by the fluorescence intensity of incorporated propidium iodide. Each graph is derived from representative experiments, and data were collected from at least 10,000 events. Independent experiments were replicated three times.

Treatment with 100 μg/mL EVP reduced the proportion of PDGF-BB-treated VSMCs in the G2/M phase to approximately 11.29% and increased the number of cells in the G0/G1 phase (Figure 2A). In addition, EVP treatment for 24 h reduced the PDGF-BB-induced cell cycle progression in a dose-dependent manner (Figure 2B). The number of PDGF-BB-induced VSMCs in the G0/G1 phase decreased after 24 h. In EVP-untreated cells, the percentage of PDGF-BB-induced VSMCs that progressed to the G2/M phase increased by 34.61%. However, EVP treatment inhibited the entry of cells into the G2/M phase in a dose-dependent manner (25 µg/mL: 29.07% ± 1.10, 50 µg/mL: 14.14% ± 0.25, 100 µg/mL: 11.52% ± 5.65).

### 3.3. Hourly Effect of EVP on the Cell Cycle in PDGF-BB-Induced VSMCs

**Figure 3:**
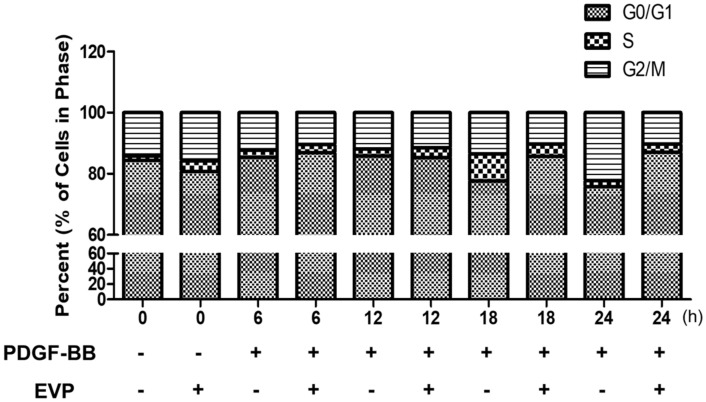
Hourly effect of EVP on the cell cycle in VSMCs induced by PDGF-BB. The cells were pre-cultured in serum-free medium for 22 h and then treated with 100 μg/mL of EVP in serum-free medium for 2 h. After treating with EVP, VSMCs were stimulated with 50 ng/mL PDGF-BB. After 0, 6, 12, 18 and 24 h, the quantity of nuclear DNA was measured in each sample based on the fluorescence intensity of incorporated propidium iodide. Each graph is derived from representative experiments, and data were collected from at least 10,000 events. Independent experiments were replicated three times.

We conducted flow cytometry on PDGF-BB-induced VSMCs treated with 100 μg/mL EVP to investigate cell cycle regulation over time. After 18 h of PDGF-BB treatment, the ratio of cells in the S phase was increased (7.64% ± 1.04) in the control VSMC group, along with DNA synthesis, and the cell cycle proceeded to the G2/M phase at 24 h (22.76% ± 4.30). In the EVP-treated group, cell cycle arrest was confirmed after 24 h (82.61% ± 0.81), in a greater proportion of cells compared with that in the EVP-untreated group (Figure 3). Therefore, we confirmed that EVP inhibited proliferation throughout the cell cycle over 24 h and reduced cell cycle progression in a dose-dependent manner.

### 3.4. Effects of EVP on the Expression Levels of Cell-Cycle-Related Proteins

**Figure 4:**
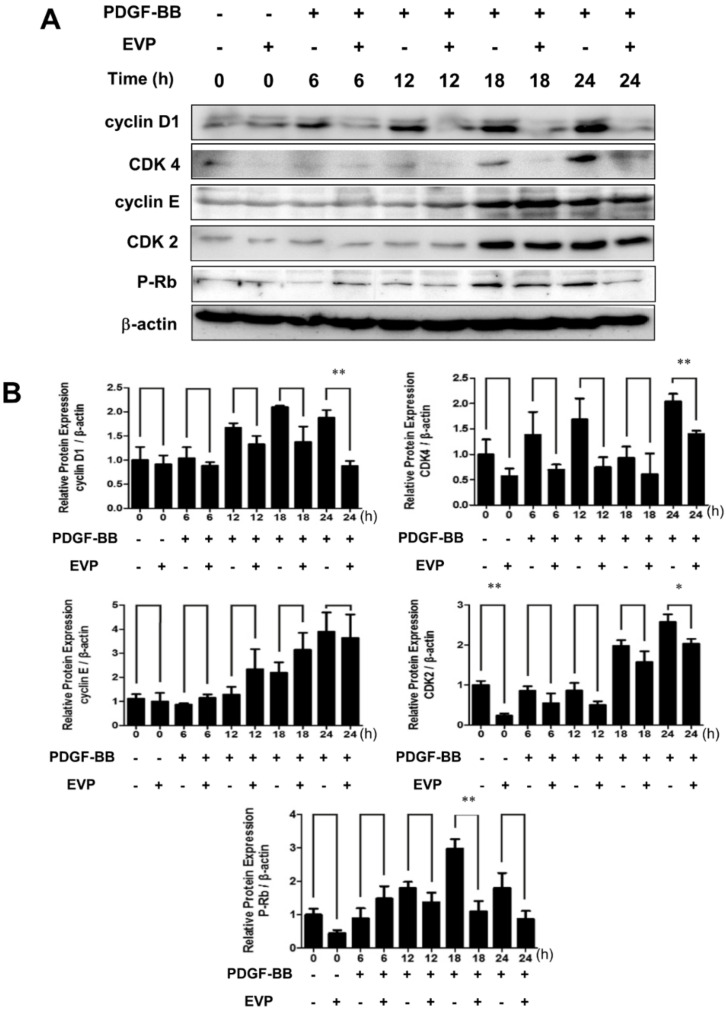
Effects of EVP on PDGF-BB-induced cell-cycle related-proteins in VSMCs. The cells were pre-cultured in serum-free medium for 22 h then treated with 100 μg/mL EVP in serum-free medium for 2 h. VSMCs were then stimulated with 50 ng/mL PDGF-BB for 24 h. (**A**) The effect of EVP on cell cycle regulatory proteins. Cells were lysed and proteins were analyzed by SDS-PAGE. Western blotting was performed with antibodies against cyclin D1, CDK4, cyclin E, CDK2 and P-Rb. β-actin was used as a control. (**B**) Graphs depicting the effect of EVP on cell cycle regulatory proteins. The graphs represent the relative expression of these proteins from three independent experiments. * *p* < 0.05, ** *p* < 0.01 compared with the EVP-untreated group.

To identify the mechanism by which EVP caused cell cycle arrest, the effects of EVP on the expression levels of cell-cycle-related proteins were investigated. Cyclin/CDK complexes have phase-specific expression levels to regulate each cell cycle phase [23]. In the cell cycle of PDGF-BB-induced VSMCs, the relative levels of cyclin D1, CDK4, CDK2 and p-Rb were increased to 1.88 ± 0.27, 2.27 ± 0.52, 2.57 ± 0.33 and 2.97 ± 0.30-fold, respectively (Figure 4). In comparison, 100 μg/mL EVP treatment decreased the relative expression levels of cyclin D1, CDK4, CDK2 and p-Rb to 0.88 ± 0.18, 1.88 ± 0.57, 2.03 ± 0.21 and 1.10 ± 0.53-fold, respectively, in early phases of the cell cycle in PDGF-BB-induced VSMCs.

### 3.5. Effect of EVP on PDGFR Signaling Pathway

**Figure 5:**
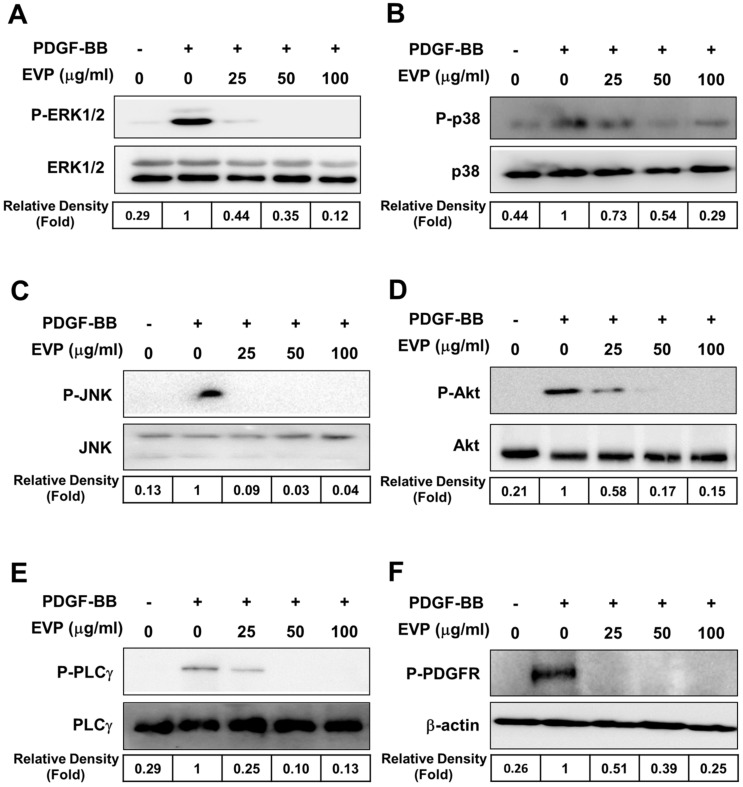
Effect of EVP on phosphorylated MAPKs and the PDGFR signaling pathway in PDGF-BB-induced VSMCs. Cells were harvested at the time the expression of each MAPK and PDGFR signaling protein was induced. Following treatment with or without EVP (0, 25, 50, and 100 μg/mL), cells were stimulated with PDGF-BB. They were then lysed in lysis buffer and Western blotting was performed to determine the expression levels of (**A**) phosphorylated ERK (15 min), (**B**) phosphorylated p38 (30 min), (**C**) phosphorylated JNK (30 min), (**D**) phosphorylated Akt (30 min), (**E**) phosphorylated PLCγ (within 10 min), and (**F**) phosphorylated PDGFR (within 5 min). Quantification of normalized densities for P-ERK, P-p38, P-JNK, P-Akt, P-PLCγ and P-PDGFR activities is shown. The images indicate the relative expression levels and inhibition of phosphorylation from three independent experiments.

The effects of EVP on the proliferation of VSMCs were also investigated. Phosphorylation of the Erk1/2, P-p38, JNK, Akt, PLCγ and PDGFRβ signaling pathways was observed in PDGF-BB-induced VSMCs. Phosphorylation of Erk1/2, P-p38, JNK, Akt, PLCγ, and PDGFRβ was increased in VSMCs induced by PDGF-BB (Figure 5). EVP significantly inhibited the phosphorylation of Erk1/2, P-p38, JNK and Akt induced by PDGF-BB in a dose-dependent manner (Figure 5A–D). Moreover, PLCγ phosphorylation was inhibited in EVP-treated cells (Figure 5E). In addition, EVP treatment decreased PDGFR-β phosphorylation in PDGF-BB-induced VSMCs. The ratio of phosphorylated proteins was 0.25 ± 0.10 for PDGFR in PDGF-BB-induced VSMCs at a concentration of 100 µg/mL of EVP compared with controls (Figure 5F). We examined the process by which phosphorylation was inhibited in PDGFR signaling pathways at various EVP concentrations. The inhibition of phosphorylation of sub-signals, including PDGFR, by EVP occurred in a dose-dependent manner, and the results demonstrate that EVP contributed to the inhibition of the PDGF-BB-induced proliferation of VSMCs.

## 4. Discussion

The World Health Organization (WHO) reported that 38% of patients died of cardiovascular disease in 2019. VSMCs are a major component of arterial blood vessels and their proliferation plays an important role in arteriosclerosis, restenosis and the onset of hypertension. Therefore, we investigated the mechanism underlying the antiproliferative effect of EVP on VSMCs as a potential prevention or treatment for cardiovascular diseases, including atherosclerosis. In the present study, we confirmed that EVP inhibited the PDGF-BB-induced proliferation and migration of VSMCs. The mechanism underlying this inhibition was found to be the regulation of cell cycle progression in VSMCs. Furthermore, the regulation of the cell cycle was confirmed by a decrease in the expression of cell-cycle-related proteins, cyclin D1, CDK4, cyclin E, CDK2 and P-Rb. It was also confirmed that proliferation was controlled by the successive inhibition of phosphorylation in the PDGFR signaling pathway, as well as the cell cycle.

The MTT assay measures the activity of living cells and has been used in many studies to measure the increase or decrease in cell number [24,25]. Lu et al. used the migration assay (wound healing assay) to obtain basic data on cell migration [26]. The results of the present study provide basic data on the inhibition of VSMC proliferation and migration by EVP in cells induced by PDGF-BB (Figure 1). The inhibition of cell cycle regulatory gene expression is, therefore, a potential method to inhibit PDGF-BB-induced VSMC proliferation [27,28,29]. Further studies on MMP9 and MMP2 in relation to the migration of VSMCs should be conducted.

The results of the present study show that the progression of the G0/G1 phase was arrested by EVP treatment in VSMCs (Figure 2 and Figure 3). EVP therefore delayed cell cycle progression into the S phase. Cell proliferation proceeds via cell cycle regulation. The cell cycle is controlled by the expression and binding of cyclin and CDKs [30,31]. The formation of cyclin/CDK complexes at each stage of the cell cycle regulates progression through each phase [32]. Cyclin D1 expression is regulated by growth factors; this cyclin is present throughout the cell cycle and is regulated in the G1 phase by CDK4 [33]. After cyclin D1 is expressed, cyclin E increases during the late G1 phase until S phase entry and forms a complex with CDK2 [34]. In the present study, EVP arrested cells in the G0/G1 phase by regulating the expression levels of cyclin D1 and CDK 4. CDK 2 expression was also inhibited (Figure 4). Thus, the complex formation with cyclin E may have been interrupted. In addition, EVP regulated cell-cycle-related protein expression in PDGF-BB-treated VSMCs, indicating that EVP treatment caused cell cycle arrest at the G0/G1 phase.

The proliferation and migration of PDGF-BB-induced VSMCs are modulated through the phosphorylation of PLCγ, AKT and MAPKs (such as p38 and Erk1/2) [35]. PLC-γ is phosphorylated upon receiving signals from the PDGF receptors [36]. Akt is regulated by the upstream PI3K signaling pathway and is involved in cell proliferation and cell cycle progression [37]. The results of the present study confirm that the phosphorylation of PLCγ, AKT, and MAPKs (such as p38 and Erk1/2) was increased by PDGF-BB (Figure 5), and this phosphorylation was suppressed by EVP in a dose-dependent manner. Thus, EVP can inhibit the PDGF-BB-induced activation of the PDGF signaling pathway and hinder VSMC proliferation and migration. Consequently, the inhibition of the PDGF signaling pathway and cell-cycle-related proteins (cyclin, CDK) by EVP treatment can be used to inhibit VSMC proliferation and migration.

## 5. Conclusions

**Figure 6:**
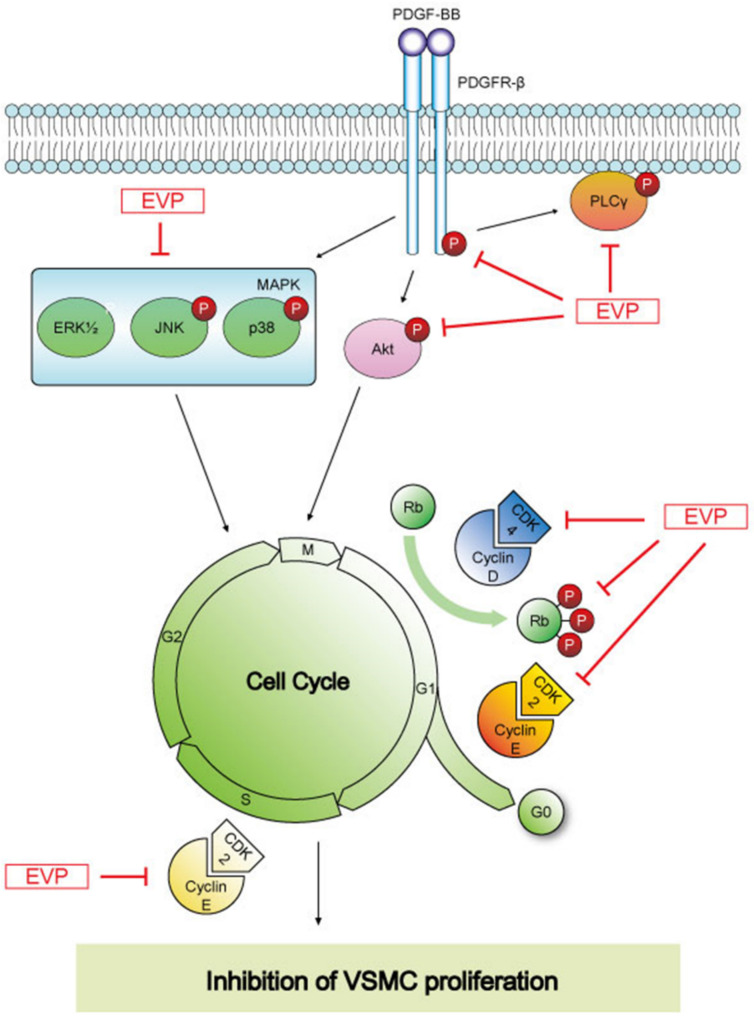
Schematic diagram of the effects of EVP on cell cycle regulatory proteins and PDGFR signaling pathway of VSMCs.

EVP extracts have been used to treat inflammatory diseases such as ear and dermal inflammation [38,39,40]. The antioxidant effects of EVP and its derivatives have been investigated previously [41,42,43]. In this study, the mechanism by which EVP affects the proliferation and migration of PDGF-BB-induced VSMCs was identified. EVP inhibited the proliferation and migration of PDGF-BB-induced VSMCs and regulated the cell cycle by blocking G0/G1 progression during VSMC proliferation. G0/G1 phase arrest was confirmed by the inhibition of cyclin D1, CDK4, cyclin E1, CDK2 and phosphorylated Rb. EVP reduced the phosphorylation levels of downstream signals, such as PLCγ, Akt, ERK, JNK and p38, through the PDGF receptor pathway (Figure 6). Our results indicate that EVP targeted the PDGF receptor pathway during VSMC proliferation. We suggest that EVP may be a regulator of abnormal cell proliferation in blood vessels induced by PDGF-BB. Therefore, EVP may prevent vascular diseases associated with VSMC proliferation and migration, and could potentially be used as a basis for the treatment of these diseases.

## Data Availability

The data used to support the findings of this study are available from the corresponding author upon request.
